# Trends in healthy working life expectancy and its difference by workload group among aged over 50 years: a longitudinal perspective

**DOI:** 10.5271/sjweh.4281

**Published:** 2026-05-01

**Authors:** Jingxuan Ma, Yuzhen Pingcuo, XiaoKe Jin, Juan Wang, Hongjian Wang, Yajia Lan

**Affiliations:** 1 West China School of Public Health, Sichuan University, Chengdu, China.; 2 Health Management Department, Chengdu Railway Center for Disease Control and Prevention, Chengdu, Sichuan, China.

**Keywords:** healthy work life expectancy, work life expectancy

## Abstract

**Objectives:**

While extending working life is a key policy objective, its impact on population health is not fully understood. This study investigated the long-term effects of physical and psychological workloads as well as initial health-work status on healthy working life expectancy (HWLE), working life expectancy (WLE), and total life expectancy (TLE) at age 50.

**Methods:**

Data were drawn from the Health and Retirement Study covering 1992–2022. The study population consisted of US adults aged ≥50 years. We implemented a multi-state life table approach based on continuous-time Markov models. Transition intensities between health and employment states were modeled to derive HWLE. Analyses were stratified by physical and psychological workload levels across three temporal cohorts.

**Results:**

Over the study period, WLE increased significantly for both sexes, while TLE slightly declined. Conversely, HWLE decreased substantially across all groups and health states. Individuals in high physical workload groups experienced shorter WLE and HWLE compared to low workload groups. High psychological load was associated with a lower proportion of healthy working years, particularly among those with initial health limitations.

**Conclusions:**

The extension of working lives is occurring at the cost of healthy years. Physical and psychological workloads exert distinct but equally detrimental effects on the sustainability of a healthy working life. These findings underscore the urgent need for targeted workplace interventions to protect worker health, particularly for vulnerable groups in high-stress or physically demanding jobs.

Population aging has emerged as an irreversible global demographic trend, significantly driven by substantial increases in life expectancy. According to the United Nations World Population Prospects 2024, global average life expectancy at birth has risen dramatically from 46.4 years in 1950 to a projected 73.5 years in 2025 (1). This demographic shift is profoundly reshaping the global labor force, leading to an increasing average age (2). The International Labor Organization (ILO) projects that workers aged 55–64 will comprise 16% of the global workforce by 2025, marking a substantial rise of 6 percentage points since 2000 (3). To address demographic pressures, raising the statutory retirement age has become the prevailing strategy. Germany is raising its retirement age to 67 by 2029; Denmark has set a target of 70 for those born after 1971. The US has raised its full retirement age to 67 for those born in 1960 or later in response to population aging as adults aged ≥65 are projected to reach 21.7% of the population by 2040 (4). Policy initiatives in the US have shifted toward extending working lives and promoting healthy aging, as evidenced by delayed retirement credits that increase benefits for delayed claiming, tax incentives for employers hiring older workers, and occupational health programs targeting age-related work limitations (5–7). However, these policies focus on extending the quantity of working years rather than the quality. Whether older workers can sustain prolonged employment while maintaining good health remains uncertain. This mismatch between policy incentives and health-based work capacity highlights the critical need to accurately assess the sustainability of the aging workforce through healthy working life expectancy (HWLE) (8).

Traditionally, working life expectancy (WLE) has served as a core indicator for assessing productive engagement. Defined as the total expected number of years an individual can participate in paid work, WLE is instrumental for calibrating pension and labor-market policies for an aging workforce (9, 10). However, WLE alone masks critical variation in the health quality of those working years. To address this limitation, HWLE refines the concept by representing the years worked in a state of good health, specifically free from chronic diseases that limit work ability (11). Using these two measures allows us to distinguish between the quantity and quality of working years, revealing whether extended employment comes at the expense of health (12).

Existing research on WLE and HWLE has consistently highlighted significant disparities across gender, socioeconomic status and different industries (13, 14). This difference is evident in China: using CHARLS data (2011–2018), studies show that at age 50, men have an average HWLE of 8.06 years compared to 5.77 years for women. Occupational differences are even starker: agricultural laborers experience nearly twice the unhealthy working life expectancy (UHWLE) as enterprise employees (15, 16). Moreover, agricultural and rural workers often remain employed well beyond the typical onset of health limitations, with women past age 65 and men close to 70 despite health problems commonly emerging around age 60 (17). Research from England provides further evidence: the HWLE for those in higher occupational classes was found to be 21–27% longer than that of their counterparts in lower classes (18).

Differences in HWLE and total life expectancy (TLE) across industries are driven by occupational characteristics. While the negative effects of work factors on WLE are well-documented, HWLE remains a significant research gap. Furthermore, few studies directly juxtapose the consequences of physical and psychological workloads, preventing a clear understanding of their relative importance. A German prospective cohort study found that middle-aged workers exposed to high physical demands experienced accelerated health decline, increasing early work exit risk (19). Similarly, a Danish cohort study found that heavy physical work reduces WLE, with men and women losing an average of 1.7 and 2.0 years by age 65, respectively (20). Nordic population data further confirm that manual occupations are consistently associated with shorter WLE compared to non-manual roles (21). Studies found that occupational stressors negatively impact work lifespan. In a Swedish cohort, high job control, which is known to buffer work-related stress, was associated with longer WLE (22). In a UK study of adults aged ≥50, low workplace support, a key determinant of work stress, was associated with an HWLE that was approximately 1.66 years shorter than among those with high support, shedding light on how occupational stress may reduce both WLE and HWLE (23). Study based US National Longitudinal Survey, psychological distress, particularly depressive symptoms, was associated with a substantial reduction in WLE, with losses ranging from 5–17 years compared to individuals reporting milder symptoms (24).

While workload is a broad concept, this study focuses on two distinct types of workload: physical and psychological. Physical load, involving tasks like heavy lifting and repetitive movements, leads to physical decline such as musculoskeletal issues (25, 26). In contrast, psychological load, driven by factors like time pressure and low job control, is a primary cause of mental health disorders and burnout (27, 28). Despite a lot of evidence on the individual consequences of physical and psychological workloads, their relative impacts on a worker’s lifespan remain largely unexplored through direct comparison. This represents a critical gap, limiting our comprehension of how these two distinct forms of work differently shape HWLE and WLE, and how these relationships have evolved historically. This study aims to measure the magnitude of HWLE and WLE losses attributable to physical versus psychological loads across three decades (1992–2022). These intervals were selected to capture distinct shifts in labor market policies and economic environments. By isolating these distinct effects, we aim to determine whether the erosion of working life is predominantly driven by workload, thereby filling a critical knowledge gap and informing the development of more targeted workplace health policies.

## Method

### Data source

**Figure 1:**
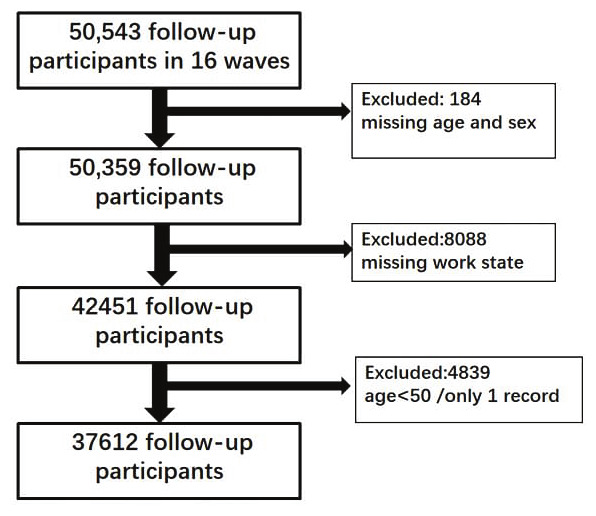
Study flow and screening process for research subjects

We analyzed data from the Health and Retirement Study (HRS), a nationally representative longitudinal cohort of the US adults aged ≥50 years launched by the University of Michigan in 1992 (29). With biennial follow-ups, the cohort has completed 16 waves of surveys from 1992 to 2022, all of which were used in the present analyses to track participants over the full 30-year period. The screening process for research subjects is shown in figure 1.

### Inclusion and exclusion criteria

Participants were eligible for inclusion if they met the following criteria: (i) were aged ≥ 50 years at baseline; (ii) had complete and coherent data on their employment history; (iii) had complete and coherent data on their medical history; and (iv) participated in at least two follow-up assessments, with follow-up time censored at the time of death or loss to follow-up.

The exclusion criteria were as follows: (i) individuals who provided logically inconsistent or incoherent responses regarding their employment information; (ii) individuals who did not participate in any paid labor during the follow-up period (eg, retirees, homemakers, or others not in the labor force); and (iii) individuals with missing data on essential demographic variables and employment information or key health information.

### Measures

Health status was defined as the absence of self-reported chronic disease and disability. Chronic conditions comprised hypertension, diabetes, chronic respiratory disease, heart disease, stroke, neuropsychiatric disorders, and rheumatism. Respondents who reported none of the listed health conditions were classified as healthy. Respondents were classified as healthy if they reported no diagnosis, or if they reported a diagnosis but indicated that the condition was “currently normal” and well-controlled. The distribution of health status calculated based on this method can be found in supplementary material (www.sjweh.fi/article/4281), table S1. Current work status was determined using two questions: (i) “What is your current employment status? Are you working now, temporarily laid off, unemployed and looking for work, disabled and unable to work, retired, a homemaker, or something else?” and (ii) “Are you currently doing any work for pay?” Individuals who answered “working now” to the first question and “yes” to the second were classified as employed. Cross-classifying health and work states yielded four mutually exclusive categories: healthy and working (HW), unhealthy and working (uHW), healthy and not working (HuW), and unhealthy and not working (uHuW).

Workload was assessed through a structured interview covering both physical and psychological dimensions. Physical workload was evaluated using three items measuring physical effort, heavy lifting, and stooping/kneeling/crouching frequency. Participants rated the frequency of these demands on a 4-point scale (1=none or almost none of the time; 4=all or almost all of the time). A total score was computed by summing the three items, with participants subsequently classified into low, medium, or high physical load groups based on tertiles of the score distribution. Psychological workload was assessed with the single item, “My job involves a lot of stress,” rated on a 4-point Likert scale. This variable was dichotomized for analysis, where “strongly agree” and “agree” indicated high stress, and “disagree” and “strongly disagree” indicated low or no stress.

### Statistic method

We used a continuous-time multi-state model to assess WLE and HWLE across combined health and work states. The analysis involved two stages. First, we fitted a continuous-time Markov model using the msm and elect packages in R (v. 4.4.1) to estimate transition intensities between the defined states (supplementary figure S1) (30, 31). These intensities were then applied in a multi-state life table analysis, also implemented with the elect package, to calculate total WLE and the expected years spent in each of the four states (HW, HuW, uHW, and uHuW). Supplementary figure S1 illustrates the state transition structure. Transitions between healthy and unhealthy states are bidirectional, but labor force exit occurs sequentially via the uHW state. Death is allowed only from unhealthy states to reflect disease-driven mortality in aging populations. Finally, we quantified the precision of these estimates by calculating standard errors from 1000 bootstrap replications.

To investigate the influence of workload, we conducted stratified analyses. To control for birth cohort effects, we grouped individuals by the year they turned 50, creating three cohorts for the periods 1992–2000, 2002–2010, and 2012–2022. We then examined changes in WLE and TLE within these cohorts, stratified by work health status. For the analysis of workload factors, the study population was defined as individuals aged 50 who were working at baseline. Our study was conducted in accordance with the STROBE-cohort checklist. This selection criterion, which encompassed both healthy (HW) and unhealthy (uHW) workers, was adopted to reduce selection bias that could arise from excluding individuals who had already left the workforce. Given the adequate sample size, statistical significance was determined using 95% confidence intervals (CI) (α=0.05).

## Result

Of the initial 50 543 participants, 12 931 were excluded (184 due to missing age and sex, 8088 due to missing work state, and 4839 due to being <50 years or having only one record), resulting in a final analytical sample of 37 612 individuals who contributed a total of 281 130 observations across the study period. Table 1 presents the number of state records for male and female subjects across three time periods. A notable overall trend is the steady increase in the total number of records from the first period (1992–2000, N=72 748) to the final period (2012–2022, N=109 447). Throughout the study period, the uHuW state was consistently among the most frequently recorded for both genders. Furthermore, the distribution of state records differed by sex, with varying proportions across states for males and females in the first two periods. The exact number of participants and ongoing participants for each wave can be found in supplementary table S1.

**Table 1:** Number of state records in three time periods. [HW=healthy and working; uHW=unhealthy and working; HuW=healthy and not working; uHuW=unhealthy and not working].

Period	Sex	HW			uHW			HuW			uHuW			Death		Total
		N	%		N	%		N	%		N	%		N	%	
1992–2000																
	Male	7499	22.62		5213	15.72		7173	21.64		12 343	37.23		924	2.79	33 152
	Female	6248	15.78		5568	14.06		8798	22.22		18 195	45.95		787	1.99	39 596
2002–2010																
	Male	8420	20.15		5703	13.65		7715	18.46		16 846	40.32		3098	7.41	41 782
	Female	8179	14.31		7436	13.01		10825	18.94		27 110	47.43		3603	6.3	57 153
2012–2022																
	Male	6018	13.16		9938	21.74		3458	7.56		23 028	50.38		3271	7.16	45 713
	Female	6145	9.64		12109	19		4933	7.74		36 692	57.57		3855	6.05	63 734

Table 2 provides a detailed list of the TLE, HWLE, and WLE for individuals with different status during three periods. Between 1992–2000 and 2012–2022, male TLE was observed from 28.71 years (95% CI 28.48–28.93) to 27.08 years (95% CI 26.34–27.81). Similarly, female TLE was observed from 31.25 (95% CI 31.02–31.48) years to 29.33 (95% CI 28.50–30.15) years. More notably, HWLE values were consistently and substantially shorter across almost all states and genders in the later period. This difference is most pronounced in individuals who are in an unhealthy state. For example, in the uHW state, HWLE for men reached 4.82 (95% CI 4.61–5.03) years in the first stage and reached 0.74 (95% CI 0.54–0.94) years in the last stage. Similarly, for women in the uHuW state, HWLE reached 2.93 (95% CI 2.38–3.47) years in 1992–2002 while it equals 0.34 (95% CI 0.24–0.44) years in 2012–2022.

**Table 2:** Total, working, and healthy working life expectancy at age 50 by health-work state and sex across three periods. [CI=confidence interval; HW=healthy and working; uHW=unhealthy and working; HuW=healthy and not working; uHuW=unhealthy and not working; TLE=total life expectancy (years); WLE=working life expectancy (years); HWLE=healthy working life expectancy (years)].

State at age 50		1992–2000						2002–2010						2012–2022				
		TLE		WLE		HWLE		TLE		WLE		HWLE		TLE		WLE		HWLE
		Mean (95% CI)		Mean (95% CI)		Mean (95% CI)		Mean (95% CI)		Mean (95% CI)		Mean (95% CI)		Mean (95% CI)		Mean (95% CI)		Mean (95% CI)
HW																		
	Male	30.82 (30.18–31.45)		12.18 (11.94–12.42)		8.18 (7.98–8.38)		31.90 (30.43–33.37)		15.00 (14.51–15.50)		7.42 (7.19–7.64)		35.97 (32.76–39.19)		21.15 (19.09–23.21)		7.12 (6.72–7.53)
	Female	35.56 (34.58–36.55)		11.58 (11.32–11.84)		7.07 (6.87–7.26)		36.91 (34.90–38.91)		14.97 (14.38–15.57)		6.61 (6.39–6.83)		41.34 (37.40–45.27)		22.16 (19.77–24.54)		6.53 (6.15–6.91)
uHW																		
	Male	28.43 (27.80–29.05)		10.79 (10.56–11.02)		4.82 (4.61–5.03)		27.53 (26.60–28.46)		11.98 (11.59–12.38)		2.12 (1.92–2.31)		29.04 (27.24–30.84)		14.85 (14.22–15.48)		0.74 (0.54–0.94)
	Female	32.39 (31.80–32.97)		10.30 (10.09–10.52)		4.13 (4.00–4.26)		31.60 (30.07–33.13)		11.86 (11.53–12.20)		1.73 (1.45–2.01)		32.73 (30.83–34.63)		14.81 (14.17–15.45)		0.57 (0.48–0.66)
HuW																		
	Male	31.27 (30.77–31.77)		11.77 (11.46–12.08)		8.72 (8.39–9.05)		29.33 (28.07–30.58)		9.67 (9.16–10.18)		5.71 (5.32–6.09)		28.03 (25.38–30.67)		8.29 (7.21–9.36)		3.80 (3.31–4.29)
	Female	33.69 (33.20–34.17)		8.87 (8.57–9.16)		6.71 (6.39–7.02)		31.80 (30.45–33.14)		7.13 (6.72–7.54)		4.41 (4.09–4.74)		30.55 (27.78–33.32)		5.93 (5.10–6.75)		2.96 (2.50–3.42)
uHuW																		
	Male	27.35 (26.13–28.56)		9.38 (8.78–9.98)		3.68 (3.10–4.27)		26.89 (24.96–28.81)		8.48 (7.82–9.15)		1.21 (0.96–1.46)		26.95 (23.52–30.38)		8.48 (7.48–9.49)		0.56 (0.40–0.72)
	Female	30.65 (29.28–32.02)		7.73 (7.18–8.28)		2.93 (2.38–3.47)		30.49 (28.33–32.65)		6.81 (6.25–7.37)		0.76 (0.57–0.95)		30.74 (26.92–34.56)		6.85 (6.02–7.68)		0.34 (0.24–0.44)
Total																		
	Male	28.71 (28.48–28.93)		10.58 (10.42–10.73)		6.29 (6.17–6.41)		27.28 (26.89–27.68)		11.34 (11.13–11.55)		4.64 (4.51–4.78)		27.08 (26.34–27.81)		13.02 (12.59–13.45)		3.91 (3.73–4.10)
	Female	31.25 (31.02–31.48)		8.86 (8.73–8.99)		4.63 (4.53–4.73)		29.69 (29.25–30.14)		9.97 (9.78–10.16)		3.56 (3.46–3.66)		29.33 (28.50–30.15)		11.90 (11.47–12.32)		3.27 (3.11–3.43)

In contrast to the lower HWLE observed in the later period, WLE for the total population was higher in the later period. For males, WLE stood at 10.58 (95% CI 10.42–10.73) years in 1992–2002, lower than the 13.02 (95% CI 12.59–13.45) years observed in 2012–2022. Similarly, for females, WLE was 8.86 (95% CI 8.73–8.99) years in the earlier period, compared to 11.90 (95% CI 11.47–12.32) years in the latest period. This indicates that although individuals spend more years at work, the proportion of working life spent in good health was lower in the latest period. In addition, substantial differences were observed by health-work state; individuals in the HW state consistently exhibited the highest TLE, HWLE, and WLE, whereas those in the uHuW state exhibited the lowest. Sex differences were observed across all three indicators. The results for all three indicators are shown in supplementary table S3 and table 2.

Table 3 presents HWLE, and WLE stratified by physical load level, health-work state, and sex across three periods. A consistent downward trend over time was observed for all three periods. From 1992–2000 to 2012–2022, HWLE generally got shorter across all subgroups, while WLE showed a consistent upward trend. For example, those with low physical load and HW state, exhibited longer HWLE at 9.67 (95% CI 9.36–9.98) years in first period, compared to 7.20 (95% CI 6.98–7.41) years in last period. Similarly, their WLE at 14.76 (95% CI 14.38–15.13) years, was lower compared to 23.82 (95% CI 21.70–25.94) years. Regarding physical load, the data reveal a significant trade-off. During the first period, among males in the HW state, those exposed to low physical load exhibited a HWLE of 9.67 (95% CI 9.36–9.98) years, in contrast to the 7.76 (95% CI 7.30–8.23) years observed for those exposed to high physical load. According the result that WLE in the high physical load group remained consistently lower than in the low load group. Specifically, in the first period (1992–2000), WLE for males in the HW state was 14.76 (95% CI 14.38–15.13) years under low physical load, compared to 10.51 (95% CI 10.23–10.78) years under high physical load. During the most recent period from 2012 to 2022, among males in the HW state, those exposed to low physical load exhibited a WLE of 23.82 (95% CI 21.70–25.94) years, in contrast to the 15.89 (95% CI 14.54–17.23) years observed for those exposed to high physical load. Individuals in the HW state consistently demonstrated a significant health advantage, with higher HWLE and HLE than those in the uHW state across all periods and physical loads. Higher physical load is linked not only to shorter healthy working years but also to a shorter total working life. Both sexes showed similar inverse associations between physical load and both HWLE and WLE.

**Table 3:** Working, and healthy working life expectancy under different physical loads in three periods. [CI=confidence interval; HW= healthy and working; uHW= unhealthy and working; HuW= healthy and not working; uHuW= unhealthy and not working; TLE= total life expectancy (years); WLE= working life expectancy (years); HWLE= healthy working life expectancy (years)].

State at age 50 and physical load		​1992–2000						2002–2010						2012–2022				
		HWLE		WLE		HWLE		HWLE		WLE		HWLE		HWLE		WLE		HWLE
		Mean (95% CI)		Mean (95% CI)		%		Mean (95% CI)		Mean (95% CI)		%		Mean (95% CI)		Mean (95% CI)		%
HW, Male																		
	Low	9.67 (9.36–9.98)		14.76 (14.38–15.13)		65.5		8.37 (8.16–8.58)		17.70 (17.08–18.31)		47.3		7.20 (6.98–7.41)		23.82 (21.70–25.94)		30.2
	Moderate	8.52 (8.20–8.83)		12.51 (12.26–12.77)		68.1		7.28 (7.06–7.49)		14.66 (14.22–15.09)		49.7		6.18 (5.93–6.43)		19.56 (17.95–21.17)		31.6
	High	7.76 (7.30–8.23)		10.51 (10.23–10.78)		73.8		6.70 (6.30–7.09)		12.00 (11.57–12.44)		55.8		5.74 (5.36–6.12)		15.89 (14.54–17.23)		36.1
uHW, Male																		
	Low	5.61 (5.29–5.92)		13.62 (13.20–14.03)		41.2		4.88 (4.69–5.08)		15.21 (14.60–15.82)		32.1		4.17 (3.96–4.37)		17.86 (15.10–20.62)		23.3
	Moderate	2.89 (2.65–3.12)		11.21 (10.95–11.47)		25.8		2.52 (2.34–2.71)		12.46 (12.02–12.91)		20.2		2.18 (1.99–2.36)		15.04 (13.28–16.80)		14.5
	High	1.69 (1.30–2.07)		9.06 (8.77–9.35)		18.7		1.48 (1.19–1.78)		10.02 (9.57–10.47)		14.8		1.28 (1.01–1.55)		12.38 (10.96–13.80)		10.3
HW, Female																		
	Low	8.21 (7.94–8.47)		13.82 (13.47–14.18)		59.4		7.02 (6.83–7.20)		17.21 (16.54–17.88)		40.8		5.96 (5.75–6.18)		24.26 (21.92–26.60)		24.6
	Moderate	7.40 (7.14–7.67)		11.49 (11.25–11.73)		64.4		6.28 (6.08–6.49)		14.05 (13.56–14.53)		44.7		5.32 (5.09–5.54)		19.74 (17.92–21.55)		27.0
	High	6.98 (6.56–7.39)		9.47 (9.20–9.73)		73.7		6.01 (5.66–6.36)		11.38 (10.91–11.84)		52.8		5.15 (4.79–5.51)		15.95 (14.45–17.45)		32.3
uHW, Female																		
	Low	4.61 (4.36–4.86)		12.86 (12.49–13.22)		35.8		3.97 (3.80–4.13)		15.10 (14.54–15.66)		26.3		3.35 (3.18–3.52)		19.92 (17.41–22.44)		16.8
	Moderate	2.46 (2.27–2.64)		10.42 (10.16–10.68)		23.6		2.12 (1.98–2.27)		12.24 (11.83–12.66)		17.3		1.81 (1.66–1.96)		16.53 (14.79–18.26)		10.9
	High	1.73 (1.36–2.11)		8.31 (8.04–8.58)		20.8		1.48 (1.21–1.75)		9.76 (9.34–10.18)		15.2		1.26 (0.99–1.52)		13.59 (12.20–14.99)		9.3

Table 4 delineates the trends in HWLE, WLE, and the proportion of life spent in healthy work (HWLE%) across three periods stratified by sex, psychological load. A principal finding is the marked and consistent decline in HWLE% across all subgroups over the three decades. This trend occurred alongside a general increase in total WLE, indicating a growing disproportion between the length of working life and the duration spent in good health. The detrimental impact of high psychological load is evident, as individuals with high load consistently exhibited lower HWLE% than their counterparts with low load. For instance, among male healthy workers, HWLE% changed from 64.0% (1992–2000) to 39.8% (2012–2022) under low psychological load, compared to a decline from 58.4% to 36.6% under high psychological load. A similar pattern, though with generally lower absolute values, was observed among females.

**Table 4:** Working, and healthy working life expectancy under different psychological loads in three period. [HW= healthy and working; uHW= unhealthy and working; HuW= healthy and not working; uHuW= unhealthy and not working; TLE= total life expectancy (years); WLE= working life expectancy (years); HWLE= healthy working life expectancy (years)].

State at age 50 and psychological load		​1992–2000						2002–2010						2012–2022				
		HWLE		WLE		HWLE		HWLE		WLE		HWLE		HWLE		WLE		HWLE
		Mean (95% CI)		Mean (95% CI)		%		Mean (95% CI)		Mean (95% CI)		%		Mean (95% CI)		Mean (95% CI)		%
HW, Male																		
	Low	8.02 (7.85–8.19)		12.53 (12.34–12.73)		64.0		6.99 (6.80–7.18)		14.57 (14.30–14.83)		48.0		7.00 (6.66–7.33)		17.58 (17.03–18.14)		39.8
	High	7.18 (7.02–7.33)		12.29 (12.10–12.48)		58.4		6.22 (6.06–6.38)		14.33 (14.07–14.59)		43.4		6.38 (6.07–6.69)		17.45 (16.85–18.04)		36.6
uHW, Male																		
	Low	5.22 (4.96–5.47)		11.08 (10.74–11.41)		47.1		2.93 (2.69–3.16)		13.27 (12.80–13.74)		22.1		2.17 (1.73–2.61)		19.31 (17.85–20.78)		11.2
	High	4.35 (4.13–4.56)		10.51 (10.21–10.82)		41.4		2.40 (2.21–2.58)		13.01 (12.55–13.46)		18.4		1.88 (1.52–2.25)		19.89 (18.26–21.51)		9.5
HW, Female																		
	Low	6.67 (6.52–6.82)		11.56 (11.38–11.74)		57.7		6.01 (5.84–6.17)		13.86 (13.58–14.13)		43.4		6.37 (6.03–6.71)		17.21 (16.62–17.80)		37.0
	High	5.92 (5.78–6.05)		11.34 (11.16–11.53)		52.2		5.37 (5.22–5.51)		13.67 (13.40–13.94)		39.3		5.85 (5.56–6.15)		17.16 (16.56–17.76)		34.1
uHW, Female																		
	Low	4.38 (4.16–4.60)		10.60 (10.28–10.92)		41.3		2.47 (2.27–2.67)		13.28 (12.75–13.81)		18.6		2.03 (1.61–2.45)		20.76 (18.92–22.59)		9.8
	High	3.61 (3.44–3.79)		10.08 (9.80–10.37)		35.8		2.04 (1.89–2.19)		13.14 (12.62–13.65)		15.5		1.79 (1.44–2.14)		21.58 (19.68–23.49)		8.3

The divergence was particularly stark for those in uHW. Under high psychological load, the proportion of healthy working life for this vulnerable group plummeted in the most recent period, reaching 9.5% for males and 8.3% for females. This represents a substantial erosion of healthy working years among the most vulnerable workers. Collectively, these findings underscore the compounding adverse effects of poor initial health status and elevated psychological stress on the sustainability of healthy work participation over time. The aforementioned trend changes can be seen in supplementary figures S2 and S3.

## Discussion

This study provides a detailed examination of the relationship between work and health in an aging workforce, revealing a significant and diverging trend. Over the past three decades, we observed a consistent increase in WLE for the general population, yet this was accompanied by a substantial and universal decline in HWLE and HWLE%. Our findings suggest that current labor market trends may be extending working lives at the cost of late-life health. A central contribution of this research is the elucidation of how specific workloads and pre-existing health statuses are relating this complex dynamic.

A key finding is the distinct role of physical workload in shaping these outcomes. In the first period, compared to those with low physical load, men in the HW state with high physical load had a WLE that was 4.25 years shorter and an HWLE that was 1.91 years shorter. This phenomenon has also been observed in previous studies, such as in Finland and Denmark, where manual workers and those exposed to physical workload factors had the lowest WLE (20, 32). This trade-off can be explained by two interrelated mechanisms. First, from a socioeconomic perspective, individuals in physically demanding occupations often face financial constraints that limit their ability to retire early, compelling them to remain in the workforce despite declining health (33, 34). Second, the accumulated physical damage from this work, which appears as musculoskeletal, cardiovascular, and other chronic conditions, directly shortens both health span and overall life expectancy. This finding is consistent with extensive research on the long-term health consequences of manual workers (35–37).

The detrimental impact of psychological workload was unequivocal, systematically reducing the health quality of work. In the first study period, high psychological stress reduced HWLE for healthy males by 0.84 years, a figure consistent with the 0.8–1 year reduction documented before (38). For individuals already in uHW state, high stress decimated their ability to work compared those in HW state. By the final period, the proportion of remaining work life spent in good health plummeted to just 9.5% for males and 8.3% for females. This translates to a mere 1.88 healthy working years for men and 1.79 years for women in this group. The trend is also consistent with previous studies (39). This synergistic negative effect, where the compounding burdens of poor health and high stress exhaust an individual’s capacity for healthy work, aligns with the health-resource depletion model and established work stress frameworks. It underscores that psychological stress is not just a minor inconvenience but a major driver of health inequality in the aging workforce. This aligns with the effort–reward imbalance model, and is further supported by a substantial body of research linking work stress to adverse outcomes like absenteeism, sickness absence, even suicide, all of which can shorten an individual’s HWLE (28, 40, 41).

Furthermore, our results reveal a widening health inequality gap. The healthy worker effect was evident in the first period as males in the HW state enjoyed 3.36 more years of HWLE than those in the uHW state. However, the most alarming finding is the collapse of HWLE for the uHW group, whose healthy working capacity has virtually vanished. HWLE plummeted from 4.82 years to merely 0.74 years for men and from 4.13 years to 0.57 years for women. Crucially, this decline occurred alongside a increase in WLE, highlighting that extended working lives are being sustained at the expense of health. This pattern indicates that the extension of working lives in this vulnerable group has come almost entirely through additional years of work in poor health, not through improved health or prolonged healthy employment. In other words, people are not becoming healthier, they are working longer despite worsening health. This raises serious concerns about policies that uniformly extend retirement ages without addressing the health constraints faced by workers in physically or psychologically demanding roles.

From a policy perspective, these findings demand a paradigm shift. Current strategies focused on delaying retirement are insufficient as they fail to account for the health costs uncovered here. Policies must move beyond a singular focus on the length of working life and urgently prioritize its health quality. This necessitates a multi-pronged approach: (i) implementing stronger workplace interventions to reduce physical and psychological loads, especially for older workers in high-demand jobs; (ii) providing targeted support and accommodations for workers with chronic health conditions to enable them to remain in the workforce healthily; and (iii) integrating health metrics, such as HWLE, into national labor and aging statistics to monitor progress more effectively. Ultimately, without addressing the fundamental issues of work quality and health inequality, the pursuit of longer working lives risks creating a future of extended years burdened by poor health, an unsustainable outcome for individuals and society.

### Advantages

This study possesses several notable strengths. First, its foundation on a longitudinal cohort with an extensive follow-up period provides a robust basis for tracking secular trends and investigating long-term trajectories in HWLE and WLE over three decades. Second, the data were collected by trained personnel using standardized protocols, which enhances the reliability and validity of the information. Importantly, our access to detailed data on both physical and psychological workloads allowed for an in-depth study of their different effects on healthy aging, an area often overlooked in large-scale research. Additionally, the large sample size across the three periods gave our study considerable statistical power, making our estimates more precise and the observed trends more reliable.

### Limitations

Despite its strengths, this study is subject to several limitations. First, data on health conditions, work status, and occupational workload exposures were entirely self-reported, with no objective verification from medical records or workplace assessments. This reliance on self-reporting introduces potential recall bias and may lead to misclassification of exposure and outcome variables. Besides, our assessment of physical and psychological workloads were based on questionnaire responses, which are inherently subjective and may not fully capture the objective, quantitative exposure. Future research can incorporate more objective measures of workload, such as ergonomic assessments or wearable sensor data, to validate and refine these findings. Third, our analysis mainly focused on sex and workload, but many other factors could also influence HWLE, WLE, and TLE. These include socioeconomic status, education, occupational class, and lifestyle behaviors. Because our models did not include these variables, our findings should be seen as providing valuable initial clues rather than a complete explanation. Future studies should aim to include these diverse factors to build a more complete predictive model. Additional limitations should be noted. The “working/non-working” classification was crude: it was assessed only annually and did not capture intermediate states such as sick leave, partial retirement, or career breaks, which may lead to an overestimation of actual time spent in paid work.

### Concluding remarks

This study shows that while WLE has increased, the proportion of those years spent in good health has declined substantially. We identify distinct mechanisms driving this trend: physical workload is associated with shorter total and healthy working lives, while psychological stress disproportionately erodes health among workers already in poor health. This creates a widening gap between healthier and less healthy workers, which risks being exacerbated by uniform policies to extend working lives. These findings challenge the assumption that longer working lives are inherently beneficial and highlight the limitations of one-size-fits-all approaches to aging workforce policy.

## Supplementary material

Supplementary material

## Data Availability

All datasets generated and/or analyzed during this study are publicly available, and detailed information can be found on the HRS official website. All data requests will be reviewed by the authors to ensure that the sharing of data does not violate the privacy of the participants or any legal agreements. Access to the data will be granted to qualified researchers for the purpose of replicating procedures or for other scholarly purposes, provided that they agree to the terms of a data access agreement.
