# Supplementary material for: Trends in healthy working life expectancy and its difference by workload group among aged over 50 years: a longitudinal perspective
Source: Scand J Work Environ Health. 2026 Apr 30;52(3):282–91. doi: 10.5271/sjweh.4281 (PMC13184823; doi:10.5271/sjweh.4281)
Supplement: Supplementary material [file SJWEH-52-282-S001.pdf]

Trends in healthy working life expectancy and its difference by workload group among aged over 50 years: a longitudinal perspective[1]

by Jingxuan Ma, PhD, Yuzhen Pingcuo, MD, XiaoKe Jin, MD, Juan Wang, MD, Hongjian Wang, MD, Yajia Lan, PhD [2]

1. Supplementary material
2. Correspondence to: Lan Yajia, Prof, West China School of Public Health, Sichuan University, Chengdu, China, 610041. [E-mail: lanyajia@sina.com]

Supplementary Figure 1 The state transition path diagram employed in this study

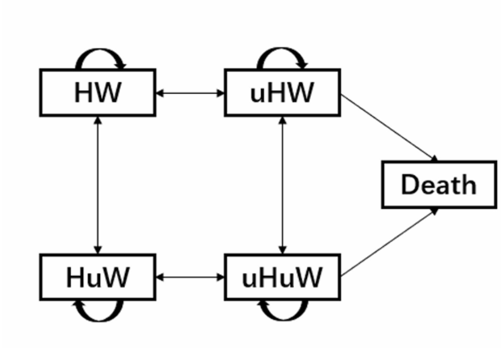

Note: HW: Healthy and Working, HuW: Healthy and Not working, uHW:Unhealthy but Working, uHuW: Unhealthy and Not working.

Supplementary Figure 2 Change in the proportion of HWLE under different physical loads over three decades

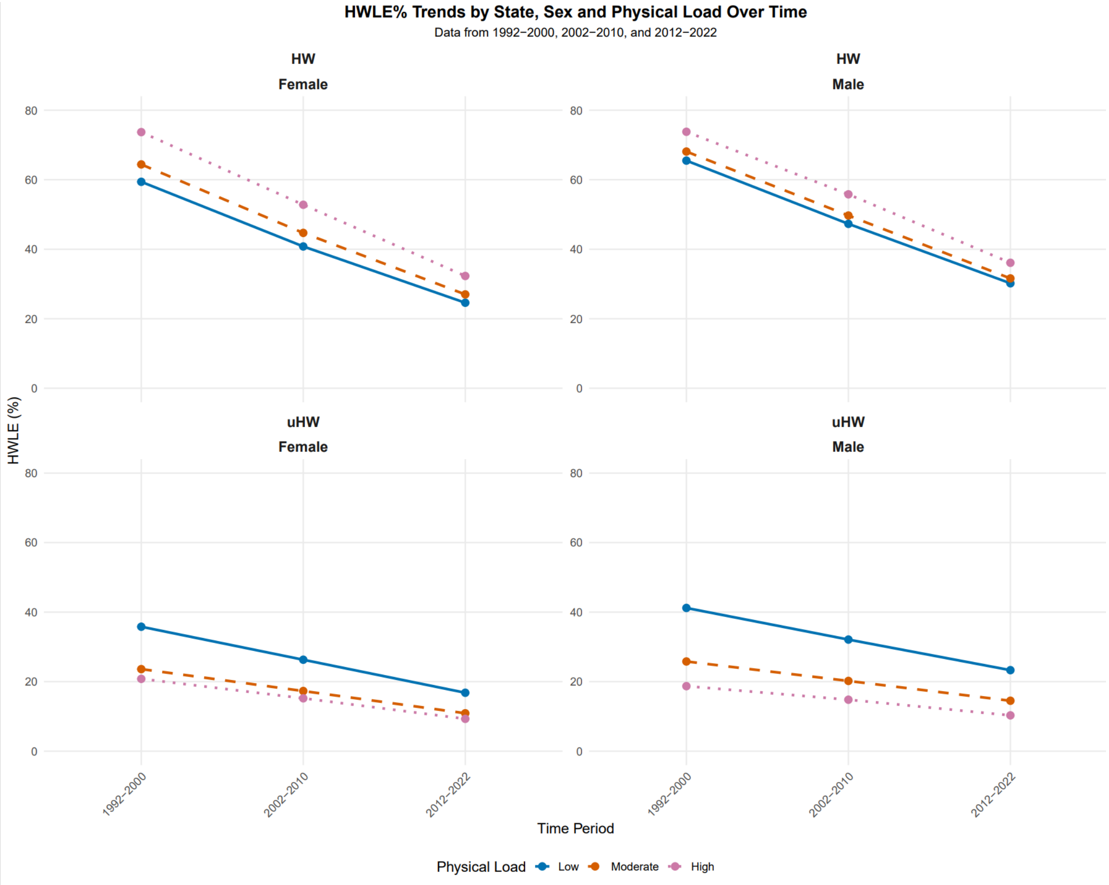

Note: HW: Healthy and Working, uHW: Unhealthy but Working, HWLE%: proportion of life spent in healthy work

Supplementary Figure 3 Change in the proportion of HWLE under different psychological loads over three decades

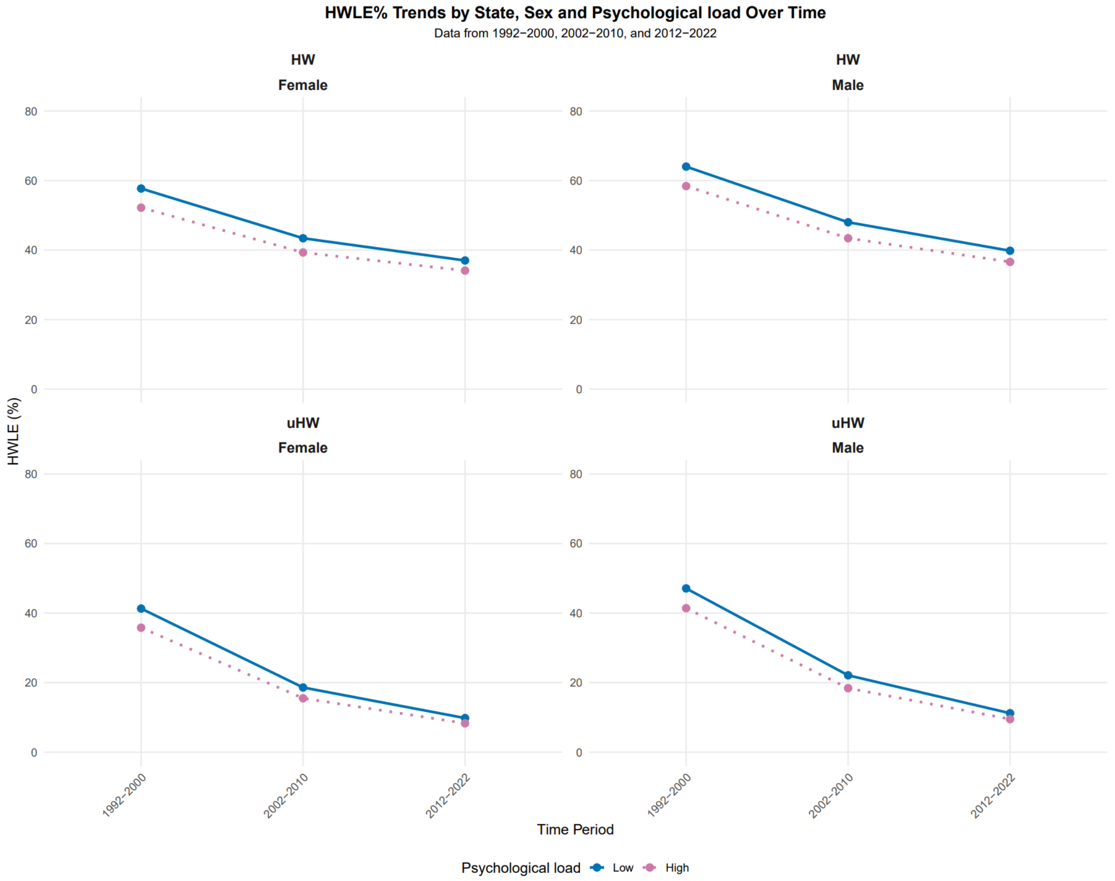

Note: HW: Healthy and Working, uHW: Unhealthy but Working, HWLE%: proportion of life spent in healthy work

Supplementary Table 1. Healthy Condition Among Survey Participants by Period (1992–2022)

Period	Unhealth		Health		Total
	N	Percent(%)	N	Percent(%)	
1992–2000	41319	56.8	31429	43.2	72748
2002–2010	57095	57.7	41840	42.3	98935
2012–2022	81767	74.7	27680	25.3	109447

Supplementary 1 summarizes the healthy conditions among participants across three survey periods (1992–2022). Chronic condition prevalence remained stable between 1992–2000 (56.8%; n=41 319) and 2002–2010 (57.7%; n=57 095) but increased substantially to 74.7% (n=81 767) in 2012–2022. Notably, the absolute number of participants without chronic conditions declined by 11.9% (from 31 429 to 27 680) despite a 50.4% growth in total sample size. Survey weights were applied for representativeness; percentages reflect within-period distributions.

Supplementary Table 2-1. Cross-Sectional Participation Distribution Across 16 Survey Waves

Wave	Participants	Participants Percentage (%)	Cumulative Number
1	1340	3.6	37613
2	3395	9	36273
3	3367	9	32878
4	4711	12.5	29511
5	2581	6.9	24800
6	2724	7.2	22219
7	4532	12	19495
8	1692	4.5	14963
9	1894	5	13271
10	2687	7.1	11377
11	1302	3.5	8690
12	1387	3.7	7388
13	2029	5.4	6001
14	875	2.3	3972
15	983	2.6	3097
16	2114	5.6	2114

Supplementary Table 2-1 reveals high attrition in early waves (Wave 1: 3.6% of total ever-participants) and variable re-engagement thereafter. Notably, Wave 4 captured peak participation (12.5%), while cumulative eligible respondents declined from 37 613 to 2 114 over 30 years. Enrollment peaked at wave 4 (12.5%; n=4 711), with cumulative attrition reducing the cohort to 5.6% (n=2 114) by wave 16. Notable resurgences occurred at waves 7 (12.0%) and 13 (5.4%), offsetting attrition from earlier waves.

Supplementary Table 2-2. Annual Longitudinal Retention Rates Among Participants (1992–2022)

Survey Year	Total Participants	Repeat Participants	Repeat Participants Proportion(%)
1992	12244	0	0
1994	11491	11422	99.4
1996	10900	10833	99.39
1998	21011	10399	49.49
2000	19483	19134	98.21
2002	18097	17854	98.66
2004	19858	16606	83.62
2006	18370	18178	98.95
2008	17129	16995	99.22
2010	21534	15526	72.1
2012	20273	19990	98.6
2014	18494	18349	99.22
2016	19945	16176	81.1
2018	16753	16612	99.16
2020	15333	15228	99.32
2022	12747	12747	100

According to Supplementary Table 2-2, retention exceeded 98% in 9 of 16 years, with temporary declines during cohort expansions (1998: 49.5%; 2010: 72.1%; 2016: 81.1%). The 100% retention in 2022 reflects final-wave completion of the core cohort. Consistently high retention (>95% in non-expansion years) validates longitudinal trend reliability.

Supplementary Table 3 HWLE, WLE, and TLE over 30 Years by Initial Health State at Age 50

State at Age 50	1992–2000			2002–2010			2012–2022		
	HWLE	WLE	TLE	HWLE	WLE	TLE	HWLE	WLE	TLE
HW	7.58 (7.43,7.74)	11.83 (11.64,12.02)	32.59 (32.03,33.15)	7.01 (6.83,7.19)	14.99 (14.52,15.45)	33.35 (31.91,34.79)	6.84 (6.47,7.22)	21.80 (19.72,23.89)	37.06 (33.89,40.23)
uHW	4.19 (4.06,4.33)	10.38 (10.18,10.58)	30.64 (30.07,31.20)	2.31 (2.17,2.45)	12.77 (12.40,13.14)	31.32 (29.80,32.84)	1.56 (1.27,1.85)	17.71 (15.93,19.49)	32.87 (29.82,35.92)
HuW	7.54 (7.28,7.80)	10.03 (9.79,10.26)	32.54 (32.16,32.92)	4.99 (4.66,5.32)	8.35 (7.91,8.79)	30.69 (29.37,32.02)	3.37 (2.88,3.85)	7.29 (6.28,8.30)	29.14 (26.30,31.97)
uHuW	2.11 (1.89,2.34)	4.90 (4.68,5.11)	26.95 (26.63,27.27)	0.99 (0.80,1.18)	3.46 (3.25,3.67)	24.41 (23.86,24.96)	0.45 (0.24,0.65)	2.41 (2.10,2.71)	22.72 (21.63,23.80)
Total	5.29 (5.20,5.37)	9.54 (9.43,9.64)	30.08 (29.90,30.25)	4.02 (3.92,4.11)	10.56 (10.40,10.73)	28.53 (28.15,28.91)	3.55 (3.39,3.72)	12.46 (12.05,12.87)	28.21 (27.43,28.99)
